# Approach to the Patient: Management of Pituitary Hormone Replacement Through Transition

**DOI:** 10.1210/clinem/dgac129

**Published:** 2022-03-09

**Authors:** Manuela Cerbone, Harshini Katugampola, Helen L Simpson, Mehul T Dattani

**Affiliations:** London Centre for Paediatric Endocrinology and Diabetes at Great Ormond Street Children’s Hospital and University College London Hospitals, London WC1N 1EH, UK; Section of Molecular Basis of Rare Disease, Genetics and Genomic Medicine Programme, University College London Great Ormond Street Institute of Child Health, London WC1N 1EH, UK; London Centre for Paediatric Endocrinology and Diabetes at Great Ormond Street Children’s Hospital and University College London Hospitals, London WC1N 1EH, UK; Section of Molecular Basis of Rare Disease, Genetics and Genomic Medicine Programme, University College London Great Ormond Street Institute of Child Health, London WC1N 1EH, UK; Section of Molecular Basis of Rare Disease, Genetics and Genomic Medicine Programme, University College London Great Ormond Street Institute of Child Health, London WC1N 1EH, UK; Department of Diabetes and Endocrinology, University College London Hospitals NHS Trust, London NW1 2BU, UK; London Centre for Paediatric Endocrinology and Diabetes at Great Ormond Street Children’s Hospital and University College London Hospitals, London WC1N 1EH, UK; Section of Molecular Basis of Rare Disease, Genetics and Genomic Medicine Programme, University College London Great Ormond Street Institute of Child Health, London WC1N 1EH, UK

**Keywords:** hypopituitarism, transition, hormone replacement, adolescence

## Abstract

Hypopituitarism in childhood is a rare, complex disorder that can present with highly variable phenotypes, which may continue into adult life. Pituitary deficits can evolve over time, with unpredictable patterns resulting in significant morbidity and mortality. Hypopituitarism and hypothalamic dysfunction may be associated with challenging comorbidities such as obesity, learning difficulties, behavioral issues, sleep disturbance, and visual impairment. Transition is the purposeful planned movement of adolescents and young adults with chronic conditions from child-centered to adult-oriented health care systems with a shift from parent- to patient-focused care. To achieve effective transition within a health care setting, the inherent challenges involved in the evolution from a dependent child to an independent adult must be recognized. Transition is a critical time medically for patients with hypopituitarism. Complex issues with respect to puberty, attainment of optimal stature, adherence to treatment, and acceptance of the need for life-sustaining medications need to be addressed. For health care professionals, transition is an opportunity for reassessment of the pituitary deficits and the need for lifelong replacement therapies, often against a background of complex psychological issues. We present 4 illustrative cases of hypopituitarism of differing etiologies with diverse clinical presentations. Diagnostic and management processes from clinical presentation to young adulthood are discussed, with a particular focus on needs and outcomes through transition.

**Learning objectives**

- Describe the causes and understand the dynamic nature of hypopituitarism.
- Describe the range of possible outcomes after transition and reflect on the management strategies in childhood and adolescence to prevent the onset of clustering comorbidities in adulthood.
- Discuss the optimal timing of transition and possible causes of delay.
- Appreciate the importance of psychological/behavioral aspects and the need to develop a holistic approach to the management of hypopituitarism.

## Hypopituitarism and Transition—Introduction

Hypopituitarism is a rare and complex endocrine disorder associated with significant morbidity and mortality (1, 2). It is highly heterogeneous, and clinical manifestations range from isolated pituitary hormone deficiency to a complete loss of all pituitary hormones with/without associated extrapituitary abnormalities. Hypopituitarism may present in the neonatal period, throughout childhood and adolescence, and in adulthood. Table 1 summarizes the main causes of hypopituitarism in childhood. Suprasellar intracranial tumors and their treatments are the main cause of hypothalamopituitary (HP) dysfunction (3-6). Congenital hypopituitarism can arise from abnormal development of the hypothalamus and pituitary and can present in isolation or as part of a syndrome in which abnormalities in structures sharing a common embryologic origin with the pituitary gland (eg, eye, midline, and forebrain) also occur (7). This in turn may be a manifestation of pathogenic variants in any of the genes involved in pituitary development (7-9).

**Table 1.** Causes of hypopituitarism in childhood

Congenital^*a*^^,^^*b*^
Isolated pituitary hormone deficiencies
Combined pituitary hormone deficiencies (≥ 2 pituitary hormonal deficiencies)
Syndromic forms of hypopituitarism (eg, septo-optic dysplasia, holoprosencephaly, severe eye defects, neck and cerebellar abnormalities, midline facial defects)
Acquired
Intracranial tumors
Craniopharyngiomas
Optic pathway gliomas
Pituitary adenomas
Germ cell tumors
Treatments for intracranial tumors
Surgery
Conventional radiotherapy
Proton beam therapy
Langerhans cell histiocytosis
Intracranial cysts
Traumatic brain injury
Infectious and autoimmune processes
Granulomatous diseases
Iron overload states
Vascular causes
Psychosocial deprivation

^*a*^Mutations in genes involved in the hypothalamopituitary development are detected in < 10% of congenital cases.

^*b*^Usually associated with structural hypothalamopituitary abnormalities on magnetic resonance imaging.

Transition is defined as the time between the completion of puberty and the achievement of peak bone mass (10). It is a time when physical changes occur, including growth and sexual maturation, as well as pronounced cognitive and emotional development. Young people begin to navigate adult relationships, employment, university education, independent living, and a social life, which may include alcohol and recreational drugs. It is a complex stage of life that can be difficult even for healthy individuals, and even more so for patients with chronic health conditions.

Transition is not always a smooth process. Table 2 summarizes some of the possible barriers to successful transition. There may be anxiety around changing health care teams and differing expectations about the roles and responsibilities of the adult compared with pediatric endocrinologists. There may also be concerns about giving autonomy to the young person with concomitant parental loss of responsibility. These issues may take time to resolve.

**Table 2.** HRT vs OCP: barriers to successful transition

HRT	OCP
Prescription charges (sometimes 2)	Free
Cyclical	Not cyclical
28 days	21 d then a 7-d break
Better for E2-dependent tissues (endometrium, bone)	Can take continuously or 3-4 breaks/y especially in young women
Transdermal and oral	
Can use patches when inducing puberty	Oral
Can have E2 separate from P	
Can give P separately (eg, E2 patch and Mirena coil)	Both in the same preparation
Need additional contraception if sexually active and reproductive age	Contraceptive
Stigma?	May be more similar to peer group

Abbreviations: E2, estradiol; HRT, hormone replacement therapy; OCP, oral contraceptive pill; P, progesterone.

We present 4 illustrative cases of hypopituitarism with different underlying etiologies and with diverse clinical presentations. Diagnostic and management processes from clinical presentation to young adulthood are discussed, with a particular focus on the needs of the patients and outcomes through transition.

## Case 1

### Overview

The patient had congenital hypopituitarism resulting from *POU1F1* (*PIT1*) mutation and was diagnosed with anterior pan-hypopituitarism (deficiencies in GH, TSH, ACTH, LH/FSH). Retesting at the time of transition revealed biochemical evidence of GH and TSH deficiencies only, resulting in a review of the initial diagnoses and rationalization of treatments.

### Learning Points

1. Pitfalls in diagnosis—Deficits in patients at high risk of evolving pituitary endocrinopathies may have been diagnosed clinically with suboptimal biochemical confirmation. The result of inappropriate overtreatment in childhood/adolescence can result in later morbidity.
2. A genetic diagnosis may provide an opportunity to challenge the diagnoses of endocrinopathies and existing treatment.
3. Retesting of the HP axis may be indicated at any stage, and transition may provide a timely opportunity to do this.

### What Could Have Been Done Differently?

Mutations in *POU1F1* are known to result in a phenotype of GH, TSH, and prolactin deficiencies (usually severe) with a small or normal anterior pituitary (AP) on magnetic resonance imaging (MRI). A genetic diagnosis in this case could have prompted an earlier review of the initial diagnoses of ACTH deficiency and central hypogonadism and retesting of these axes. Transition provided an opportunity for reevaluation of endocrinopathies in this case but the sequelae of this was a change in diagnoses and treatment at an already fragile time.

### Case Summary

Birth weight showed from -1 to 21 SD score (SDS); the patient’s ethnicity was Afro-Caribbean. At birth, he was found to have bilateral undescended testes, wide anterior fontanelle and sagittal suture, uptilted nose, and short limbs. He had normal developmental milestones. Central hypothyroidism was incidentally diagnosed at 1.4 years in the context of an admission for a hypoglycemic seizure and a history of poor growth. Severe short stature (-7.4 SDS) was noted. Clonidine testing showed undetectable GH. Prolactin was repeatedly very low. MRI scans showed a hypoplastic AP. The patient was started on levothyroxine (L-T4) after proving cortisol sufficiency, and on GH replacement with an excellent growth response. He was retested at 6.5 years: cortisol peak after glucagon stimulation was suboptimal at 335 nmol/L with reported subtle symptoms of hypocortisolism (tiredness). He was therefore started on hydrocortisone. The patient had subsequent development of hyperphagia and weight gain. At 11.83 years, he was noted to be prepubertal with some psychological distress and evidence of growth deceleration and was therefore started on testosterone replacement without any further testing, given the presence of other pituitary deficiencies, previous cryptorchidism, and small AP on MRI. A de novo heterozygous mutation (c.811C > T, p.R271W) was subsequently detected in the gene encoding *POU1FI* (*PIT*1) (11), accounting for his severe GH, TSH, and prolactin deficiencies; however, this could not explain the putative ACTH and gonadotropin deficiencies (8).

**Figure 1.**
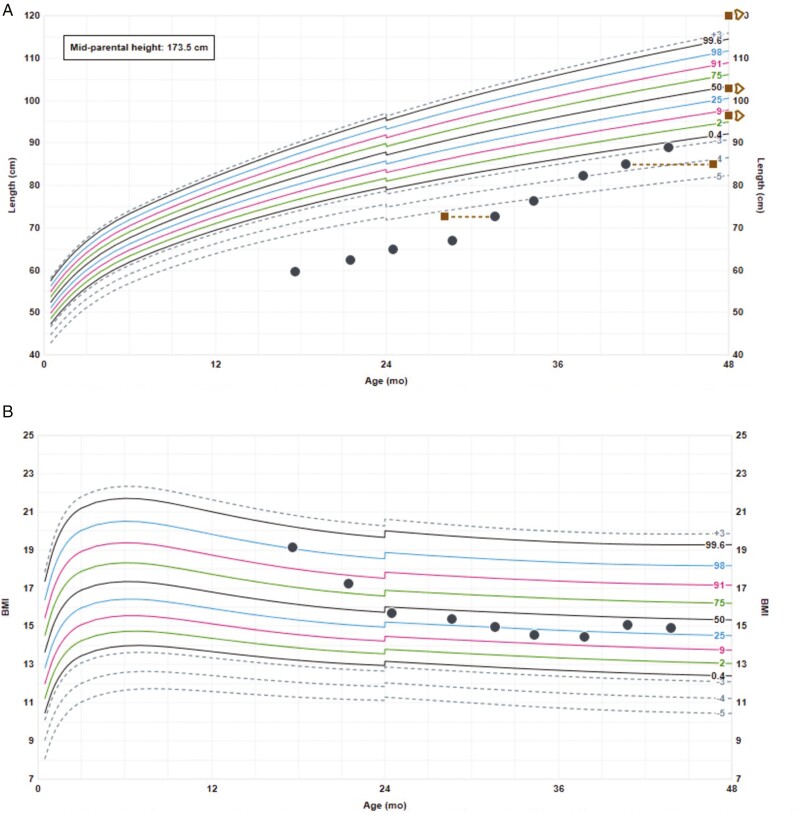
Growth chart—case 1. (A) Height; the dotted lines represent the bone ages. (B) Weight. (C) BMI. BMI, body mass index.

A discussion about transition was started at 15.75 years, with transfer to the adolescent clinic at 16.4 years and then to the adult services. His final height was significantly impaired (153.7 cm, -3.12 SDS) (Fig. 1) with obesity (body mass index [BMI] 33.1 kg/m^2^ [+2.87 SDS]) (Fig. 1). Retesting at 18.03 years with combined provocation tests (insulin-tolerance test [ITT], LHRH, and thyrotropin-releasing hormone [TRH]), after discontinuation of his testosterone, GH, and hydrocortisone treatments, demonstrated a cortisol peak of 627 nmol/L, baseline morning testosterone of 14.3 nmol/L, and adequate gonadotropin peaks (LH 15.1 and FSH 5.8 U/L). Persistent prolactin (15 mU/L, normal range 44-548), GH (peak 0.2 mU/L), and TSH (baseline free T4 [FT4] < 5.1, blunted TSH response after TRH stimulation: T0 minutes, 0.79 mU/L; T20 minutes, 1.18 mU/L; T60 minutes, 1.07 mU/L) deficiencies were in keeping with the phenotype of *POU1FI* mutations. He therefore remains on GH and L-thyroxine replacement.

## Case 2

### Overview

The patient had acquired hypopituitarism resulting from langerhans cell histiocytosis (LCH). Delayed transition to the adult endocrine service was in part due to oncological surveillance with delayed transition to adult oncology and in part due to complex social issues and significant learning disability.

### Learning Points

1. The evolution of endocrine deficits in patients with hypopituitarism is variable in timing and severity. Central diabetes insipidus (CDI) can be the presenting manifestation, particularly from pituitary stalk lesions.
2. In children with acquired disorders, endocrine sequelae and other comorbidities can arise many years after diagnosis of an HP lesion/treatment. Hypothalamic dysfunction is particularly difficult to manage, and severe hypothalamic obesity is a common finding in acquired and congenital causes of hypopituitarism.
3. Timely transition to adult services is important, particularly for complex patients under multiple specialities, although coordination of transition of care can be more challenging. A delay in transition may result in greater morbidity.

### What Could Have Been Done Differently?

Earlier transition to adult endocrinology might have prompted earlier review of treatment options. Given the presence of multiple associated comorbidities, this patient could have been commenced on transdermal as opposed to oral estrogen replacement, although the prevailing practice at that time was to commence patients on oral estrogen.

### Case Summary

**Figure 2.**
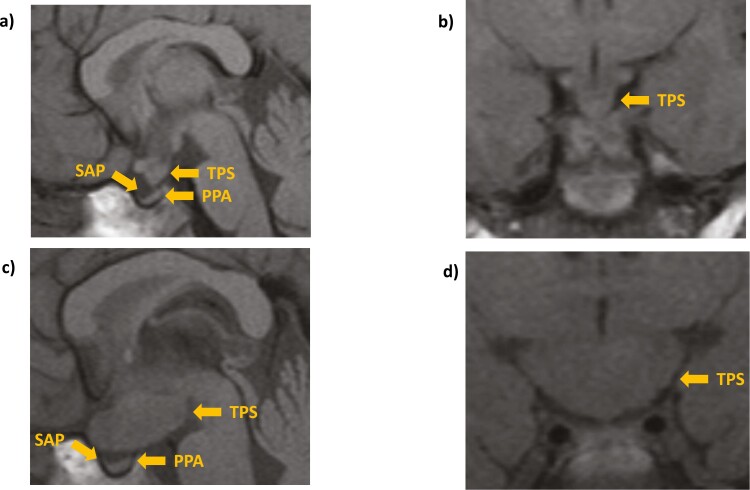
Brain magnetic resonance imaging—case 2. (A and B) Images taken at the age of 3.15 showing thickened pituitary stalk, absent posterior pituitary, and small anterior pituitary on (A) sagittal and (B) coronal views. (C and D) Images acquired at the age of 4.25 years showing marked enlargement of the suprasellar lesion on (C) sagittal and (D) coronal views. PPA, posterior pituitary absence; SAP, small anterior pituitary; TPS, thickened pituitary stalk.

Her birth weight was 0.95 SDS. Born in Iraq and moved to the United Kingdom as a refugee at the age of 2.5 years. Her clinical presentation at 2.9 years was with sudden onset of polyuria/polydipsia; CDI was diagnosed on a water deprivation test and treated with desmopressin (DDAVP). An MRI showed a thickened pituitary stalk with an absent posterior pituitary bright spot (Fig. 2A and B). TSH, ACTH, and GH deficiencies were documented. She was started on hormone replacements, apart from GH, which was withheld while the cause of thickened pituitary stalk was being investigated. Multisystem (skin, skull, and hypothalamus) LCH was diagnosed on a skeletal survey and skin biopsies. At age 4.1 years, she developed hyperphagia, rapid weight gain, labile mood, poor sleep, and recurrence of polyuria and polydipsia despite DDAVP treatment, as well as a deterioration in her vision. A second MRI showed marked enlargement of her suprasellar mass (Fig. 2C and D). A stalk biopsy revealed a fibrous lesion with no active LCH. She was treated with low-dose focal radiotherapy and steroids in an attempt to preserve her vision. Immediately after radiotherapy, her vision stabilized and sleeping/eating patterns transiently improved. However, she continued to gain weight and reported excessive somnolence. A further biopsy of her lesion aged 6.3 years showed a cellular pattern typical of juvenile xanthogranuloma; she was treated with chemotherapy with clinical improvement. This was again transient; increased appetite and weight gain continued. Metformin was commenced with minimal effect. At 9.2 years of age, after an interval of lesion stability, she was also started on GH replacement. At 14 years of age, pubertal induction was initiated with oral ethinylestradiol. This was later discontinued because of the development of iliofemoral deep vein thrombosis (DVT), and she was started on long-term anticoagulation therapy.

**Figure 3.**
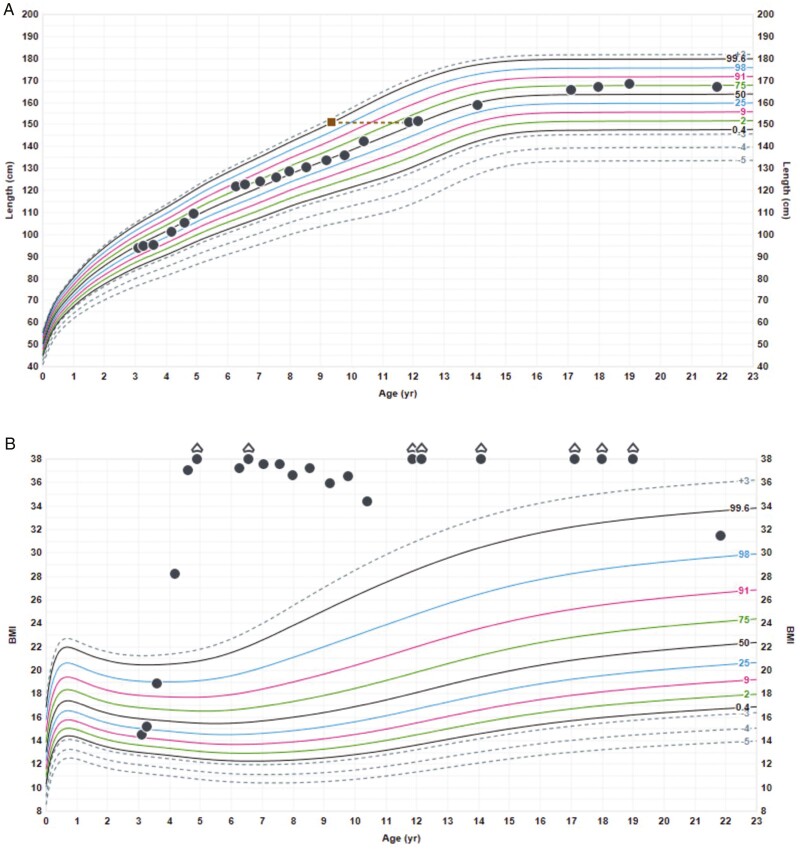
Growth chart—case 2. (A) Height. The dotted lines represent the bone ages. (B) Weight. (C) BMI. BMI, body mass index.

She attended her first transition clinic at 20.15 years and was then transferred to adult services. As a young adult, she has a low bone mineral density (BMD) (*z* score -3.1 at the lumbar spine) and severe insulin insensitivity (Homeostatic Model Assessment for insulin resistance, 9.1). Commencement of transdermal estrogen is currently under discussion given the bone health benefits. She was switched from an oral hydrocortisone dose 4 times a day to a slow-release preparation twice a day. DDAVP regimen has been readjusted from 3 to 2 times a day. Hyperphagia and weight gain remain a significant issue, with metformin and orlistat having limited effect. Weight and height at the latest follow-up (21.5 years of age) were 128 kg (+3.73 SDS) and 167 cm (+0.83 SDS) respectively, with a BMI of 45.9 kg/m^2^ (+4.07 SDS) (Fig. 3).

## Case 3

### Overview

The patient had congenital hypopituitarism resulting from septo-optic dysplasia (SOD) syndrome with associated learning difficulties, challenging behavior, and autism

### Learning Points

1. The presence of neurobehavioral disability often leads to delayed transition. It is not uncommon to see complex patients transferred late to adult services, probably compounded by a “resistance to change” by both the family/patient and the health care teams that they know well. This is compounded by a lack of adequate psychological and social support to young people at the time of transition and in adult services.
2. The process should be developmentally appropriate and individualized.
3. Decisions about management in young people lacking capacity who have complex needs are often taken in the context of best interest meetings.
4. Transition to adult services is important to enable development of experience and expertise of adult specialists in the management of rare conditions. Their practice needs to adapt to the needs of such patients with multidisciplinary team (MDT) input, frequent contact with patients, and an understanding of the important roles of the patients’ carers.

### What Could Have Been Done Differently?

Earlier transition would have enabled earlier engagement of adult services to support medical and additional needs. However, adequate MDT (nursing, psychology and social) support is critical for optimal care of the patient.

### Case Summary

**Figure 4.**
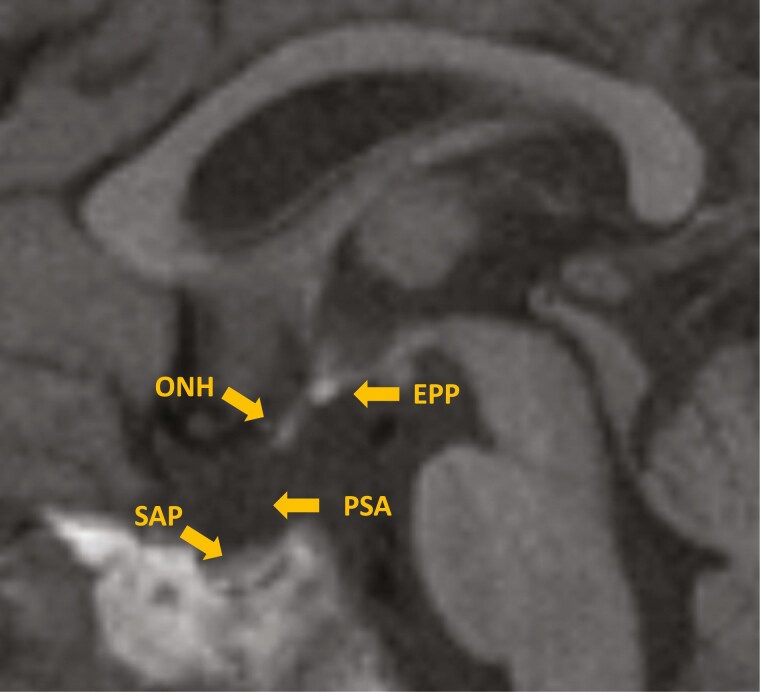
Brain magnetic resonance imaging—case 3. The image shows very small anterior pituitary, ectopic posterior pituitary, absent pituitary stalk, small optic nerves and chiasm. EPP, ectopic posterior pituitary; ONH, optic nerve hypoplasia; PSA, pituitary stalk absence; SAP, small anterior pituitary.

Born at term with no antenatal/postnatal complications to a young mother (age 17 years). At 3 months of age, the patient was diagnosed with SOD, presenting with severe visual impairment resulting from bilateral optic nerve hypoplasia and hypopituitarism (GH/TSH/ACTH deficiencies). MRI showed a very small anterior pituitary, ectopic posterior pituitary, pituitary stalk absence, and small optic nerves and chiasm (Fig. 4). He received frequent hydrocortisone dose adjustments in early infancy because of recurrent hypoglycemia. He was later diagnosed with severe learning difficulties and autism. Over the years, he developed hypothalamic syndrome with obesity, sleep disturbance, and thermoregulation issues. The weight gain was compounded by a lack of physical exercise resulting from neurobehavioral issues and sweet snacks given to treat presumed hypoglycemia. His frequent sweating episodes, initially attributed to hypoglycemia, were shown to be related to hypothalamic dysfunction (HD) because hypoglycemia was never documented. He developed severe sleep disturbance, which was partially helped by melatonin treatment. Entered puberty early (around 10 years) with a deterioration in behavior. A decision was made with the family to suppress puberty with a GnRH analogue until late adolescence. After discontinuation of the GnRH analogue at 19 years of age, pubertal progression did not restart until the age of 22 years.

Transitioned to adult services at 24.7 years. As a young adult, he developed impaired glucose tolerance. He has ongoing problems with dental abscesses, which are difficult to treat because of the need for general anesthesia. Latest auxology (24.7 years) was weight 109 kg (+2.72 SDS), height 170.7 cm (-0.93 SDS), and BMI 37.4 kg/m^2^ (+2.97 SDS). Current care requirements include adult services that can support his additional needs, support for management of adrenal insufficiency/crisis, input from other teams including mental health and social services, adult learning disability services, and weight management. Decisions about his management are often taken in the context of best interest meetings.

## Case 4

### Overview

Congenital hypopituitarism resulting from structural HP abnormalities. Reevaluation of pituitary function at the time of transition and in early adulthood resulted in discontinuation of all treatment.

### Learning Points

1. GH treatment is generally safe in childhood. Avascular hip necrosis has been rarely described in case reports of children on GH replacement treatment, although the causality is unclear.
2. Testosterone supplementation should be discontinued before performing a semen analysis, and spermatogenesis may still take some months to recover.
3. Transition may be a time of conflict between a young person and his or her parents/carers. The young person should always be directly involved if possible, and efforts should be made to facilitate the process of building independence.
4. Adolescence is often a time when poor adherence to treatment may occur. Transition may provide opportunities to simplify treatment regimens with a view to improving compliance.
5. Continued surveillance for pituitary dysfunction is warranted in the presence of a structurally abnormal pituitary MRI.

### What Could Have Been Done Differently?

Fertility issues are often only discussed after transition to adult services. These conversations should ideally start at a younger age and include discussions about relationships, body image, sexual health, and contraception. Young people should be offered a discussion with specialist fertility services.

### Case Summary

**Figure 5.**
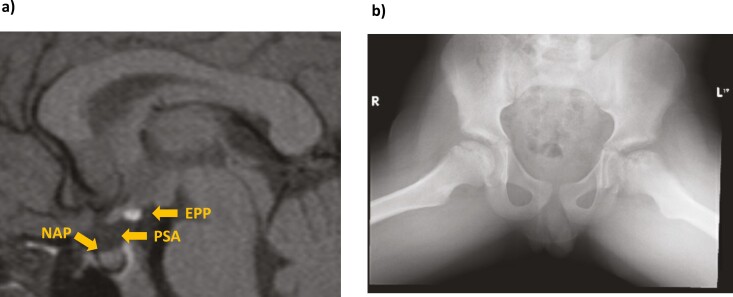
Brain and hip imaging—case 4. (A) Brain magnetic resonance imaging shows EPP, PSA, and NAP. (B) Pelvis and hips radiograph showing (i) marked flattening and sclerosis of the left femoral head in keeping with avascular necrosis, (ii) some sclerosis on the right side, and (iii) posteromedial displacement of the capital femoral epiphysis bilaterally. EPP, ectopic posterior pituitary; NAP, normal anterior pituitary; PSA, pituitary stalk absence.

**Figure 6.**
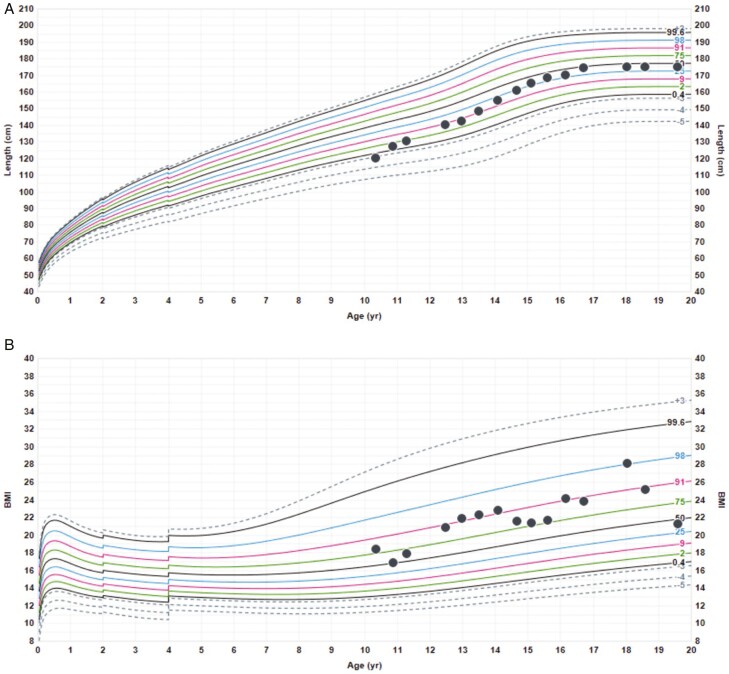
Growth chart—case 4. (A) Height. The dotted lines represent the bone ages. (B) Weight. (C) BMI. BMI, body mass index.

This patient received a late diagnosis of GH deficiency (GHD) with growth failure presenting at age 10.5 years (GH peak after glucagon 1.2 mcg/L). TSH deficiency was documented after the start of GH treatment. An MRI of the brain showed an undescended or ectopic posterior pituitary (EPP) and pituitary stalk absence (PSA) and a normal anterior pituitary (Fig. 5A). After being treated with GH for 1 year, it was then discontinued for 6 months because of the development of left avascular hip necrosis and displacement of the capital femoral epiphysis bilaterally (Fig. 5B). A femoral osteotomy was performed because of pain. GH was then restarted at a lower dose. He was prepubertal at 13.5 years, despite normal responses to GnRH and 3 days and 3 weeks of human chorionic gonadotropin. He was commenced on testosterone replacement predominantly to alleviate psychological distress. At age 16 years, he presented with fatigue and had low cortisol concentrations; hydrocortisone replacement was started. Testosterone treatment was discontinued at the age of 16.5 years because his testicular volume had increased to 20 mL bilaterally. He was retested at the age of 17.6 years: peak GH to insulin-induced hypoglycemia was 0.3 mcg/L, cortisol was 407 nmol/L, and peak LH was 20.3 IU/L. He was not keen to restart GH. He continued hydrocortisone replacement, but this was switched to prednisolone because of erratic adherence. He requested to restart testosterone replacement because of the lack of facial hair. He transferred to adult services at age 18 years and attended clinic alone and is fully independent. His final height is 176.6 cm (+0.29 SDS) with a BMI of 24.15 kg/m^2^ (-0.1 SDS) (Fig. 6). At this time, he started to question his treatments because he found it difficult to take medication regularly. This resulted in family tension with parental anxieties pertaining to adrenal insufficiency. He underwent further endocrine assessment with an ITT, which confirmed normal cortisol secretion (basal 208 nmol/L, peak 543 nmol/L) and a GH peak of 3.9 mcg/L. After a transient borderline increase in his TSH, his thyroid function also normalized off thyroxine replacement. Testosterone replacement was discontinued to assess gonadal function. Morning investigations showed testosterone 19 ng/dL, LH 4.4 IU/L, and FSH 8.8 IU/L. A first semen analysis showed no sperm; however, 6 months after the cessation of testosterone treatment, a second semen analysis showed a normal sperm count. He discontinued all hormone replacement by the age of 25 years. His BMD *z* score (lumbar spine) was -2.2. Weight-bearing exercise, dietary calcium, and vitamin D supplementation were encouraged. He has had his first child without the need for fertility treatment. He continues to have ongoing surveillance, given the presence of a structurally abnormal pituitary MRI.

## Discussion

The 4 cases presented highlight important considerations when managing young adults with hypopituitarism at the time of transition.

### Transition

Transition is the purposeful planned movement of adolescents and young adults with chronic conditions from child-centered to adult-oriented health care systems (12). The goal of transition is to provide care that is uninterrupted, coordinated, developmentally appropriate, psychosocially sound, and comprehensive (12).

During this time, young people face all the challenges associated with becoming an adult with a long-term medical condition, including talking to friends about the need for life-sustaining medications. There are patient safety issues for those with adrenal insufficiency and/or diabetes insipidus, and patient support and education is key. Simplifying treatment regimens with less frequent administrations during the day (cases 2 and 4) may improve compliance. The presence of hypogonadism may raise concerns with respect to future relationships, sexual function, body image, and fertility (case 4). Associated abnormalities such as visual impairment, obesity, autism, and learning disabilities may add to difficulties in management (case 3) and there can be challenges for those patients with additional needs accessing appropriate services in adulthood. Psychological support may be helpful.

Transition clinics with joint input from pediatric/adolescent and adult endocrinologists, as well as an endocrine clinical nurse specialist (CNS), are critical to successful outcomes. Ideally, a transition coordinator should facilitate this process. In the UK National Health Service, the National Institute for Clinical Excellence standards recommend that transition starts at about age 14 years, and young people who will move to adult services have an annual meeting to review transition planning (13). The young person should always be directly involved, and efforts should be made to facilitate the process of building independence (13). They should be offered time apart from their parents during their appointments. Ideally, clinics should be in a clinical environment that makes young people feel more at ease. Texting, email messaging, patient portals, and phone/video appointments may all help adolescent engagement, as may flexibility in clinic appointments; for example, evening appointments may suit some adolescents and those who work or are at university/college (14).

However, meeting the recommended target for the best timing of transition might not always be possible. Transition was significantly delayed (>20 years of age) in 2 of our cases. The presence of neurobehavioral disability as well as family psychosocial issues played a major role in case 3, whereas the simultaneous oncological surveillance performed at the pediatric hospital with delayed transition also by the oncology team was a contributory factor in case 2, as, again, were learning and psychological difficulties. It is not uncommon to see complex patients transferred late to adult services, probably compounded by a “resistance to change” by both the family/patient and the health care teams that they know well. For patients, it can be difficult to detach emotionally and move to the adult services. Additionally, it might be challenging to find adult services with sufficient expertise in the management of these rare conditions; many cohorts would not have survived to adulthood a few decades ago. However, although many pediatric providers and parents feel that delaying transition permits continuity of care, posttransition surveys from young adults indicate that young people felt more independent and in charge of their disease if transitioned earlier (15). It is also recognized that the process should be developmentally appropriate and take into account each young person’s capabilities, needs and hopes for the future, as well as taking place at a time of relative stability for the young person (13). Access to nursing in the form of a CNS and psychological support may not be easily available when in adult care.

### Assessment of the HP Axis

The diagnosis of hypopituitarism can be challenging, particularly in the neonatal period and in the peripubertal years. Patients may be started on hormone treatment inappropriately. This was certainly the issue in case 1, when the patient was started on hydrocortisone and testosterone unnecessarily.

Basal pituitary hormone screening performed early in the morning helps identify hypopituitarism. In some cases, dynamic testing is not needed; for instance, for the diagnosis of TSH/TRH deficiency (16), although this can be performed in the context of a combined dynamic stimulation of the HP axis in patients with suspected multiple pituitary hormone deficiencies. The necessity for a TRH test is debatable. Stimulation tests are necessary for the diagnosis of GHD and usually for that of ACTH deficiency. Similarly, a water deprivation test might be needed to diagnose CDI. With respect to gonadotrophin deficiency, a GnRH stimulation test, with GnRH-induced maximal LH cutoff of 4.3 IU/L, together with a testicular volume cutoff of 1.1 mL, and basal inhibin B concentration <35 pg/mL, have been proposed as the most effective discriminators of congenital hypogonadotropic hypogonadism (CHH) from delayed puberty in adolescent males (17). However, this test carries its own limitations (18) and must be interpreted in the clinical context. For females, LH, FSH, and estradiol, with additional anti-Mullerian hormone and antral follicle count if a patient has had gonadotoxic treatment, may be of more use. In boys, a combination of GnRH and 3 days and 19 days human chorionic gonadotropin test may aid in differentiating constitutional delay of growth and puberty and CHH (19).

MRI is warranted in all patients with documented hypopituitarism. Some structural HP abnormalities such as EPP and PSA are significantly associated with an earlier development of pituitary deficits (1, 20). MRI is essential in patients with suspected brain tumors.

The role of genetics in congenital hypopituitarism remains to be established and testing is currently offered on a research basis only. To date, pathogenic variants have been identified only in a modest proportion (<10%) of patients (7-9), suggesting a potential role for other yet-to-be-discovered genes along with environmental/epigenetic factors (21).

### Evolution of Endocrinopathies

The evolution of endocrine deficits in patients with hypopituitarism is variable in timing and severity (1). The most common isolated deficiency is GHD and the most common pattern of evolution in patients with multiple deficits is GH->TSH->ACTH (1). CDI can be the presenting manifestation of congenital hypopituitarism, particularly within the SOD spectrum, from infiltrative disorders, pituitary stalk lesions as observed in case 2, or after surgery for brain tumors (22).

Congenital hypopituitarism is a dynamic condition in which new deficits can appear years after the initial diagnosis. Children with isolated GH deficiency (IGHD) have a significant risk of developing additional pituitary deficiencies (23). This risk ranges between 5.5% in childhood-onset idiopathic IGHD and 35% in adult-onset organic IGHD (24, 25). Hypothalamo-pituitary (HP), optic nerve (ON), and midline brain abnormalities predispose to a higher risk (up to 45%-100% in children with pituitary stalk interruption syndrome) (1, 26).

In children with acquired disorders, endocrine sequelae can arise many years after radiotherapy/proton beam therapy (22). Surveillance of growth, puberty, weight, neurodevelopment, and endocrine status is recommended for at least 15 years after tumor therapy (27, 28). In 242 pediatric brain tumor survivors, followed for a mean of 6.4 (0-23.4) years, the prevalence of endocrinopathies was 72%, with GHD being the most frequent (53%), followed by central hypogonadism (22%), central hypothyroidism (15%), accelerated/precocious puberty (13%) and pubertal delay (12%), CDI (12%), and ACTH deficiency (10%) (29). Patients treated with cranial irradiation/proton beam therapy and those diagnosed at a younger age have a higher risk of developing endocrine deficits, which can develop over time (30).

### Retesting

Transition is an opportune time for reevaluation of any endocrinopathies, considering the difficulties in younger children, but also that recovery of anterior pituitary function is well described and as stated previously, new deficits can evolve. Between 25% and 88% of patients are seen to recover GH secretion at the time of transition (31-34). Retesting of GH secretion is indicated in patients with childhood-onset GHD after the completion of linear growth (growth velocity <1.5-2 cm/y). Current guidelines suggest that retesting should be performed in all patients except in those with ≥ 3 pituitary hormone deficiencies and low-serum IGF-1 concentrations (<-2.0 SDS), those with genetic defects affecting the HP axis, and those with HP structural brain defects (35). The ITT is the gold standard for the diagnosis of adult GHD, although this is contraindicated for patients with a history of seizures or heart disease. There is no agreed GH peak for young adults, and different health care systems choose a cutoff point for transition of 3 to 6 µg/L to define GH deficiency (35-37). In addition, different tests use different GH concentration to diagnose GH deficiency, and obesity can be a confounding factor (29, 32, 33). When contraindicated (patients with seizures and cardio/cerebrovascular disease), alternative tests such as the glucagon, the macimorelin, and the GHRH plus arginine/GH secretagogue tests can be used, which are well described elsewhere, although there is a lack of normative data at transition (35, 38). Whatever test is chosen, this should be undertaken at least 1 month after discontinuation of GH therapy.

In patients with multiple pituitary deficiencies who have received additional hormonal treatments during childhood, reevaluation of the need for long-term replacement therapies should also be performed.

According to current guidelines (35), case 1 with a documented *POU1F1 (PIT1)* mutation should not have been retested, but, in this case, further testing documented normal secretion of both ACTH and FSH/LH. It is clear that the initial diagnoses of ACTH and gonadotrophin deficiencies were flawed, and perhaps these replacement therapies could have been avoided during childhood. The glucagon test is not a reliable test for the diagnosis of cortisol deficiency (39) and LH/FSH deficiency was never biochemically confirmed but assumed in this patient given the delayed onset of puberty and the presence of other associated pituitary deficits, as well as the previous undescended testes. Case 4 with EPP and PSA was also able to discontinue all his treatments after retesting because he did not meet the criteria for adult GH treatment and had normal thyroid, adrenal, and gonadal function off treatment. It is known that patients with EPP (and small anterior pituitary) may normalize GH production at retesting (40), although our patient also had PSA, which is usually associated with persistent deficits (33). In keeping with current guidelines, cases 2 and 3 were not retested and their treatment regimens were readjusted in frequency and dosage.

GH treatment is generally safe in childhood. Case 4 developed hip necrosis and GH was discontinued for 6 months. Hip necrosis has been rarely described in case reports of children on GH replacement treatment (41), although the causality is unclear. It is possible that patients with GH deficiency might have preexisting orthopaedic problems (42). In patients with brain tumors, it is common practice to commence treatment after a period of lesion stability, as in case 2. American guidelines suggest waiting until the patient with a previous cancer diagnosis has been disease-free for at least 1 year and has completed any oncological treatment (43); however, there is no robust evidence supporting this timeframe. Pediatric brain neoplastic/inflammatory lesions have a tendency to recur and there is little evidence to support a causative role for GH in the etiology of recurrent or new tumors (44). In retrospect, earlier initiation and continuity of GH treatment might have improved metabolic outcomes for case 2, without significantly affecting the evolution of her underlying LCH disease.

### Hormone Replacement—Considerations

The mainstay of medical treatment in patients with hypopituitarism is replacement of the appropriate hormones.

GH is generally a safe treatment, and in tumor survivors, replacement dosages are not associated with recurrence or progression (44-46). A small increased risk of second primary neoplasms (45) has been reported in patients who have previously received radiotherapy (44). There may be an increased risk of type 2 diabetes in GH-treated patients, but this appears to be confined to those with preexisting risk factors (44). GHD should be treated until linear growth is complete, and GH secretion should then be reassessed. Most data support the role of this therapy at transition and beyond in patients with permanent GHD (35, 47-53). GH benefits during transition include improvements in peak BMD (54) and body composition (55). In adulthood, benefits are based around cardiovascular surrogate outcomes, body composition, bone density, and quality of life (QoL) (47). Monitoring should include IGF-I, lipid profile, glycated hemoglobin concentrations and QoL measures. IGF-I should be used to titrate GH dose, and this is lower than those used in childhood, (0.2-0.5 mg/d) (56). Younger patients (57), especially women on oral estrogen, may require higher doses (35) because oral estrogens cause hepatic GH resistance resulting from a first-pass effect in the liver (58, 59).

Puberty may be normal, delayed/absent, or precocious in patients with hypopituitarism, the latter being mainly encountered in patients with SOD (1) and in those with optic pathway glioma (OPG) and germ cell tumour (60).

In patients with CHH, it is not unusual to commence GH supplementation at least 6 months before sex steroid replacement. With respect to hypogonadotropic hypogonadism (HH), replacement in males is commonly administered in the form of testosterone (parenteral or transdermal). Puberty is induced slowly over approximately 2.5 to 3 years, with careful monitoring of growth, virilization, trough testosterone concentrations, and full blood count (for the adverse effect of polycythemia). Longer acting testosterone preparations (eg, 3 monthly) may be used once growth has been completed. Testosterone replacement in teenage boys induces secondary sexual characteristics, but does not induce testicular enlargement, which can be circumvented by gonadotropin treatment (61).

In girls, estrogens can be given orally or transdermally. The transdermal route is more physiological in the form of 17β-estradiol, which avoids first-pass liver metabolism. Additionally, it has fewer risks and may be the safest option in patients with a history of DVT (62, 63, 64). There is evidence to suggest that it leads to better outcomes for bone density and uterine development (63). Concentrations of genotoxic estrogens are also lower with this route (64) and there is no evidence to suggest an increase in breast cancer for young women on estrogen replacement. When puberty is being induced, the dosage is gradually increased over a period of 24 months. Monitoring growth, secondary sexual characteristics, uterine volume, and blood pressure is recommended (65). Progestogen is added once breakthrough bleeding occurs. There are different ways of taking estro-progestinic replacement after puberty has been induced, either with combined cyclical hormone replacement therapy (HRT) regimens or combined oral contraceptive pill preparations (Table 3). Transdermal HRT should be considered first because it is more physiological with 17β-estradiol. Oral HRT can then be considered first because it can be cyclical without a 1-week break each month and has lower doses of the synthetic ethinylestradiol, whereas combined oral contraceptive pills are not cyclical and contain higher doses of ethinylestradiol and progestin. However, the oral contraceptive pill is a contraceptive, and some young women may prefer this, and it can also be given continuously. It is therefore important to offer an individualized approach to estrogen replacement. In women, but not in men, late pubertal timing has been associated with higher risk for osteoporosis (66). Current guidelines suggest using dual X-ray absorptiometry scans to monitor bone density after adult HRT has been instituted (67).

**Table 3.** Barriers to successful transition: hormone replacement therapy vs oral contraceptive pill

General
Lack of preparation/training/support
Lack of documentation
Lack of formal transition booking system and formal point of contact during the process
Moving hospitals/environments
Lack of engagement from the young person
Belief that delaying transition might result in a better outcome
Dropping out
Differing needs of young adults and different perspectives of adult medical care
Greater independence of care in young adults under adult medical care with less parental supervision
Less access to educational support/social care in the adult world
Lack of involvement of a dedicated psychology service
More specific for patients with hypopituitarism
Lack of consensus with respect to optimal management
Lack of resources in terms of time and management from the multidisciplinary team to provide optimal care
Lack of consensus about the role and responsibilities of the pediatric vs adult endocrinologist
Lack of experience from adult services in managing some rare conditions (eg, septo-optic dysplasia)
Lack of capacity in patients with severe neurobehavioral disabilities
Multiple medications with reduced adherence in adolescence
Interactions between some medications and some recreational drugs
Sensitive issues with respect to fertility and sexual development
Reduced final height affecting self-esteem
Clustering of comorbidities compromising quality of life, self-esteem, and independence

TSH deficiency is easily managed with L-T4 (once ACTH deficiency has been excluded), with dose titrations based on FT_4_, and not TSH. It is generally recommended that FT_4_ concentrations should be maintained in the mid- to upper half of the normal range, although there is little evidence to support this (68).

Replacement doses of hydrocortisone for ACTH deficiency should be given at least 3 times daily in children. This regimen is needed to provide replacement that is as physiological as possible. New modified-release oral glucocorticoids have also been developed to deliver more physiological glucocorticoid replacement in childhood (69, 70). Conversely, longer acting medications (eg, corticosteroids such as prednisolone) may be chosen in adolescence and young adulthood, when growth is complete, and the priority is to improve concordance. A recent single-blind randomized controlled trial showed that a once-daily modified-release glucocorticoid replacement therapy reduced body weight and the incidence of recurrent infections and improved QoL in adults with adrenal insufficiency compared with the conventional treatment administered multiple times a day (71).

At transition, education of the young person on increasing oral glucocorticoids during intercurrent illness, how/when to administer emergency intramuscular hydrocortisone, and to correct hypoglycemia if an issue during adrenal crises, is essential (72). Input from the endocrine CNS is essential to help support the young person to understand the importance of taking their glucocorticoid replacement and to help them achieve independence. Discussion around timing of hydrocortisone replacement is important because adolescents may stay up late and wake up late. Additionally, empowering adolescents with information on how to prevent adrenal crises, as well as addressing lifestyle changes such as going out, staying away from home, and staying safe if using alcohol and drugs, may save lives. Doses should be doubled, given 6 hourly and or given parenterally 6 hourly in the face of significant illness (73). Interventions such as patients wearing medical alert jewelry, carrying a steroid emergency card, having keyring tablet holders so that they always have medication with them, and having an automatic pill dispenser with radio-controlled times can be useful for patient safety and well-being. Patient support groups and peer support using social media may be a useful source of information for young people (eg, Addison disease Self Help Group).

Management of CDI, particularly in patients with coexisting ACTH deficiency and/or hypothalamic adipsia, is complex and needs specialist care. Oral DDAVP may be preferred because it is widely available, generally well absorbed, and easier to use in patients with visual impairment and physical disability (74). Untreated patients with an intact thirst and free access to fluid are able to maintain euvolemia/eunatremia by adjusting their oral intake. DDAVP treatment aims to provide the patient with a good QoL and sleep by reducing the burden of polyuria and polydipsia. At the time of transition, young people should receive education to understand the importance of not stopping their DDAVP, carrying it with them at all times, and adopting appropriate strategies if they become unwell, particularly if they have coexisting ACTH deficiency. Recreational drugs such as MDMA (“ecstasy”) may lead to polydipsia, which could be extremely dangerous in patients treated with DDAVP. Alcohol can increase urine output, and young people should be told they will not necessarily need extra DDAVP if they drink a large amount of alcohol. In addition, some clinicians advise missing a dose of DDAVP if drinking a large volume of alcohol to prevent water overload. Again, patient support groups can be useful for information (www.pituitary.org.uk).

### Obesity and Hypothalamic Dysfunction

Despite their different etiologies (SOD and LCH), cases 2 and 3 both manifested significant morbidity caused by HD. Severe hypothalamic obesity is a common finding in acquired and congenital causes of hypopituitarism. In patients with concomitant ACTH deficiency, hydrocortisone is often blamed as a contributory factor (case 1). However, unlike other conditions such as congenital adrenal hyperplasia, in which supraphysiological doses may be required to suppress the elevated ACTH, physiological replacement doses of glucocorticoids are used in hypopituitarism. Other hormone deficiencies may contribute to weight gain and/or abnormal body composition; however, these factors are easily reversible by starting/adjusting the respective replacements. The dramatic and inexorable trajectory of weight gain associated with HD is caused by disruption of the hypothalamus leading to a combination of hyperphagia and low metabolic rate (75). Lack of exercise might also be an issue in some patients with learning disabilities and/or visual impairment (case 3).

Management of hyperphagia, obesity, and the other hypothalamic symptoms is extremely challenging. No medical treatments have been shown to achieve sustained weight loss in hypothalamic obesity although GLP-1 agonists may be of use (76). Bariatric surgery can help some patients, as described in a small cohort study by van Santen et al (77), but is probably not suitable for all patients (78). No medication is available for the other features of HD and treatment is largely symptomatic.

### Puberty and Reproductive Function

The pubertal development of adolescents with hypopituitarism often requires some support/intervention. Cases 1 and 4 illustrate the challenges in the differential diagnosis between constitutional delay of growth and puberty and CHH with FSH/LH deficiency. The 2 conditions have similar clinical and biochemical profiles in adolescence and often the definitive diagnosis can only be made in retrospect in early adulthood (18). Gonadal function might seem impaired in adolescence, but then “recovers,” even in patients with other anterior pituitary deficits. It could be argued that, despite being clinically prepubertal when puberty was expected, neither of our 2 patients had biochemical evidence of HH. Delayed puberty in adolescents can be associated with significant anxiety about body image in terms of physical size and pubertal immaturity, decreased self-esteem with social isolation, withdrawal from sporting activities, and psychosocial and peer relationship difficulties. In these circumstances, there is evidence that sex steroid therapy can be beneficial (79).

Fertility issues are often only discussed after transition to adult services (case 4). Young adults should have discussions about sexual health and fertility and be advised to use contraception if they are sexually active, even if they are diagnosed with hypogonadism. It is important to remember that CHH is not always a lifelong condition and reversibility appears to be more common (~10%-15%) than previously thought, even in patients with documented mutations (80). There are no clear factors for predicting reversible CHH. Importantly, recovery of reproductive axis function may not be permanent. Thus, periodic treatment withdrawal with close monitoring and follow-up are warranted in these patients. Patients should have access to specialist fertility services if they request this. Patients with HP disease do well with induction of spermatogenesis or ovulation induction; therefore, cryopreservation is not usually needed unless there is additional gonadal toxicity from chemotherapy for childhood cancer survivors.

Testosterone supplementation should be discontinued before performing a semen analysis. In a study of men taking testosterone for hypogonadism or infertility, 88.4% were azoospermic while on treatment, but 65% recovered spermatogenesis 6 months after its discontinuation (81). Novel pediatric therapeutic strategies have also been trialed with the aim of enhancing the sexual and reproductive function of these patients in adulthood (82). For example, in addition to their use in the induction of fertility in adults, gonadotropins can also be used to induce pubertal maturation in adolescent males and to improve genital abnormalities (ie, micropenis and cryptorchidism in neonates with HH), with the further ultimate aim of improving their future spermatogenesis and fertility (61). Although potentially beneficial, these novel treatments are still experimental.

Whether oral estrogens in replacement doses might have contributed to the development of DVT in case 2 is difficult to ascertain because several factors (including her obesity, some degree of immobility, and her underlying diagnosis of LCH with the several oncological treatments received) might also have played a role.

The onset of early puberty in case 3 posed significant management issues for the family because of the worsening of behavioral issues. In girls with learning disabilities, an early onset of periods, with attendant issues regarding hygiene and self-care, might also be difficult to manage for the family/carers. Therapeutic options are available for reduction/suppression of menstruation (83), at the same time optimizing bone mass accrual. For patients requiring estrogen replacement who do not want periods, either the combined contraceptive pill can be taken continuously or noncyclical HRT can be used.

True central precocious puberty is not uncommon in patients with SOD (1) and in those with suprasellar tumors (60). Patients undergoing treatment with parenteral GnRH analogues might experience a transient pubertal arrest after their discontinuation (lasting a few years; case 3). This may raise the possibility of the development of HH, particularly in patients with other anterior pituitary hormone deficiencies. Development of HH in patients with a previous history of central precocious puberty resulting from OPG has also been reported (46).

### Bone Health

Reduced bone density is not uncommon in adolescents and young adults with hypopituitarism. Peak bone mass occurs at the end of somatic growth. At least 90% of peak bone mass is acquired by age 18 years (84), although gains in BMD of 5% to 12% have been observed during the third decade (85). It is now clear that the diagnosis of osteoporosis should not be made solely upon BMD measurements because none of the techniques available provides information about bone mineral content, density, and architecture (86). However, these measurements are useful to monitor the patients’ progress over time (86), complementing the clinical and biochemical assessment.

Cases 2 and 4 had BMD *z* scores < -2 SDS, but no evidence of fractures. In case 2, the etiology was likely to be multifactorial from a combination of immobility, history of steroid treatment, and estrogen deficiency. Transdermal estrogen replacement might be the cardinal intervention needed in this case. In case 4, it could be speculated that the inability to receive GH treatment for 6 months in childhood, and the patient’s decision to discontinue the GH treatment after retesting, despite his low GH response at retesting later in adolescence, might also have played a role. Bisphosphonates should be used with caution in young adults, especially in young women of childbearing age because of its very long half-life (87, 88), and transition is a good time to reassess the need for this. Weight-bearing exercise (89), optimization of nutrition to ensure adequate calcium intake (https://www.bda.uk.com/resource/calcium.html), and keeping vitamin D concentrations within the normal range (90) are generally encouraged in all young adults, especially those with low bone density.

### Learning Disability and Lack of Capacity

Case 3 is an example of several challenging problems posed by patients with a combination of learning disabilities, autism, and blindness, often simultaneously present in children with SOD and in those with suprasellar brain tumors. The lack of capacity to consent to medical treatment options can make management decisions difficult. Best interest meetings frequently take place to ensure that the best treatment possible is achieved while respecting, as much as possible, personal choices and beliefs. Families are often under significant pressure as young adults may not be able to live independently and have significant additional needs; mental health teams, social care, and adult learning disabilities teams may need to be involved. Provision for these young adults is frequently woefully inadequate.

## Conclusions

Transition is a gradual process of change. Adolescents with hypopituitarism face physical and emotional changes as well as facing the prospect of a chronic condition in which lifelong hormonal treatment may be required. Expert care across the life span is required but there is no universal consensus on how transition should be provided or how the transfer of care between teams should be coordinated. This is even more challenging in countries with limited resources. It is important to give young people the knowledge and confidence to manage their condition and support them through the process. The need for a holistic approach, therefore, is never greater than at the time of transition. A multidisciplinary professional team ideally including pediatric and adult endocrinologists, psychologist, ophthalmologist, neurodevelopmental pediatrician, dietitian, CNS, adult learning disability teams, and social worker, should work in partnership with patients and their families, with the ultimate aim to ensure successful transition into adult life, and all that that encompasses.

## Data Availability

Some or all datasets generated during and/or analyzed during the current study are not publicly available but are available from the corresponding author on reasonable request.

## Abbreviations

AP: anterior pituitary
BMI: body mass index
BMD: bone mineral density
CDI: central diabetes insipidus
CHH: congenital hypogonadotropic hypogonadism
CNS: clinical nurse specialist
DDAVP: desmopressin
DVT: deep vein thrombosis
EPP: ectopic posterior pituitary
FT4: free T4
GHD: GH deficiency
HD: hypothalamic dysfunction
HH: hypogonadotropic hypogonadism
HP: hypothalamopituitary
HRT: combined cyclical hormone replacement therapy
IGHD: isolated GH deficiency
ITT: insulin-tolerance test
LCH: langerhans cell histiocytosis
MRI: magnetic resonance imaging
ONH: optic nerve hypoplasia
PSA: pituitary stalk absence
QoL: quality of life
SDS: SD score
SOD: septo-optic dysplasia
TRH: thyrotropin-releasing hormone
